# Prosthetic rehabilitation of multiple-digit amputa tions using silicone material in sub-Saharan African country Ghana

**DOI:** 10.11604/pamj.2020.36.357.25361

**Published:** 2020-08-27

**Authors:** Akouetevi Aduayom-Ahego

**Affiliations:** 1 Ahelite Brace, Accra, Ghana,; 2 Dream GP Inc., Research and Development Division, Osaka, Japan,; 3 Waseda Institute for Sport Sciences, Waseda University, Tokorozawa, Japan

**Keywords:** Africa, amputations, Ghana, hand, prosthesis, silicone

## Abstract

Digit loss might be due to traumatic injuries, congenital malformations and various diseases. Rehabilitation of finger amputations requires aesthetics and functional aspects. Due to limited resources in low income countries, some local professionals find it difficult to meet the patient´s needs. One of the methods of gaining total suspension is to don the residual finger with aesthetic silicone prosthesis. A 25-year-old female presented with multiple-digit amputations of both of her hands due to autoimmune disease. At first stage of the treatment, silicone prostheses of the loss fingers of both hands were planned. The impressions of the remaining fingers were taken to manufacture the silicone prostheses. The manufactured prostheses with total suspension improved patient´s social life. The patient´s residual fingers were fitted with the prostheses after which she was satisfied with the aesthetic aspects.

## Introduction

Amputations leave subjects to several forms of disability. Distal amputations are commonly due to work related accidents, traffic road accidents, and other sort of traumatism or diseases. This leaves a lot of stigmatization associated with psychological aspects of the patient [1,2]. In addition, the appearance and the aesthetic aspect of finger prosthesis play a significant role in patient life [3]. However, the rehabilitation of patients with finger amputation in developing countries remains very challenging for technicians due to training facilities and lack of appropriate materials. It is estimated that only 5-15% of people with disabilities can get access to assistive products in the low-resource countries [4]. Previous studies stated that only 5% of the population of people with disabilities could get access to rehabilitation services in Ghana [5]. Moreover, the silicone technology has not been introduced in many undergraduate programs in the Prosthetics and Orthotics schools. Recent studies in West Africa reported that graduates wished to introduce silicone technology in their centres [6-8]. This case report describes the rehabilitation of a patient with multiple finger amputations in Ghana, West Africa. At the time of writing this report, there was no previous published data on manufacturing of silicone finger prosthesis in the country.

## Patient and observation

**Figure 1:**
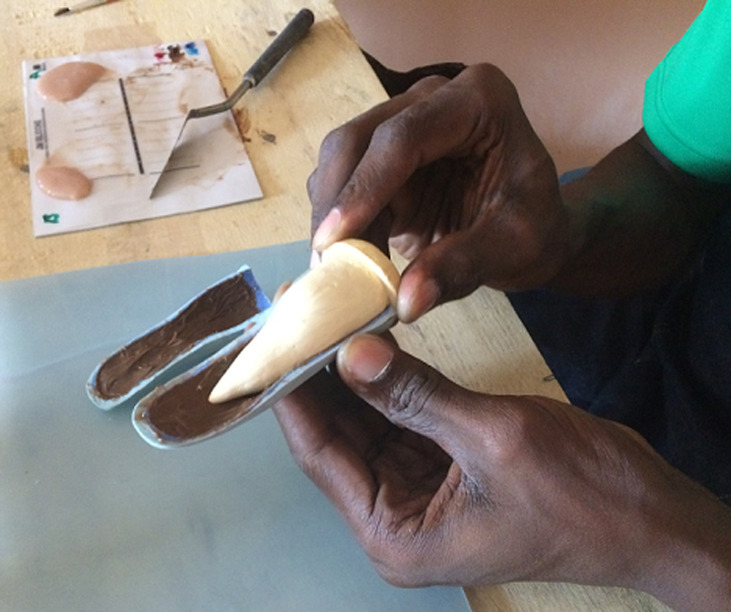
packing the silicone in the mold

**Figure 2:**
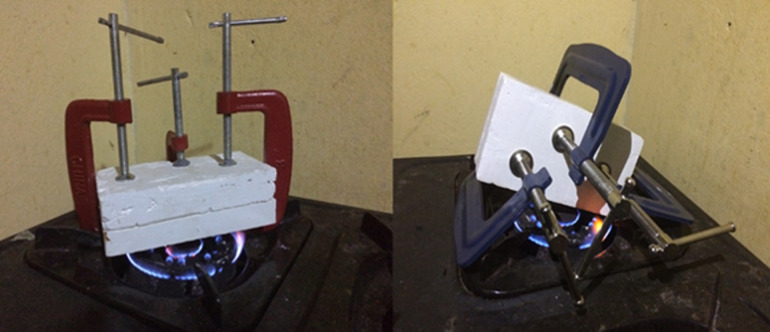
packed silicone in the mold and heating of the mold with gas flame

**Figure 3:**
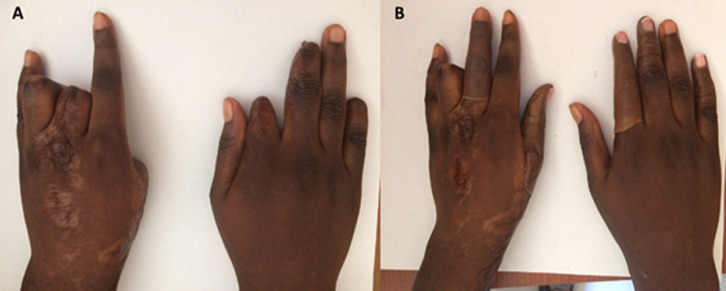
fitting of the finger prosthesis (A: before wearing the prosthesis, B: patient´s hands with the prostheses)

**Figure 4:**
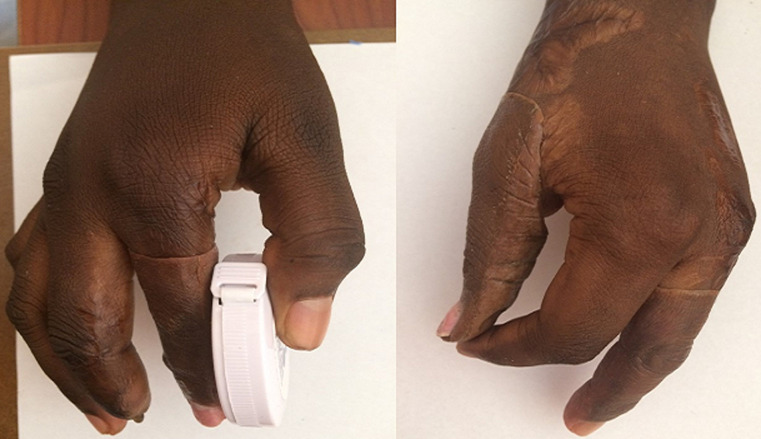
patient trying to grab an object during the fitting

A 25-year female patient visited our prosthetic office with deficiencies of both hands due to autoimmune disease. The amputations were performed in 2015. After the examination of both hands, it was revealed that in the right hand; the metacarpal of the index finger, the distal phalanges of the second and fourth fingers were amputated; and in the left hand; the metacarpals of the thumb and of the second fingers and the proximal phalange of the third finger were amputated. It was also noticed that there was a flexum of the proximal phalange of the stump of the third finger. Since the amputations, the patient has never worn any prosthesis. The subject has requested finger prostheses to improve the quality of life. The case was discussed and with the consent for the patient´s rehabilitation of fingers, silicone prostheses was planned for the first stage of the treatment. The measurements were taken by a Certified Prosthetist. The manufacturing of the prosthesis consisted of the following steps: the impression taking, the wax pattern and the laboratory works as described in former studies [9,10]. The case described here presented with multiple amputations of both hands which present the amputation of the distal phalange of the second finger as well. That made it difficult taking the impression on the sound finger. So, the impression was done on the contralateral finger of the patient. The impressions were taking with hydrophilic vinyl- polysiloxane impression material. The finger was in relax position while taking the pattern. Two layers of the impression material were wrapped on the fingers for about 10 minutes and the negative molds were taken off after the setting was done. Then the liquid wax was poured into the impression mold to retrieve the positive replica of the hand. Then another impression was taken from the wax mold to create the final mold in which the silicone solution was inserted. To ensure a total suspension of the prosthesis, a suspension cavity was created in the mold using a pin made with cast following technique described in the former study [10]. In order to match the patient´s skin colour, the artist´s oil colours were used to mix with the silicone solution. To ensure total aesthetics, the nail part was first packed then the dorsal and palmar aspects were mixed and packed separately in the mold (Figure 1). Due to unavailability of electric oven to heat the silicone solution, the mold was closed and heated using a kitchen gas stove for about an hour. In order to have all the surface of the cast with the average temperature, the surfaces of the cast mold were turned periodically on the stove gas (Figure 2). The finished prosthesis was then retrieved from the mold and excess silicone flesh was trimmed from the margins. The prostheses were tried on the patient after she was educated on how to take care of the prosthesis (Figure 3). Through the technique described above, good suspension and real looking finger prosthesis as patient´s skin colour was manufactured. The subject was able to don the prosthesis on her own and had no pain when wearing it and was able to grab an object (Figure 4). The patient was satisfied with the final aspect of the prosthesis (Figure 3, Figure 4).

## Discussion

Upper limbs rehabilitation service in Africa faces many challenges including poverty, lack of material and lack of expertise of the local staff [6,7]. Moreover, some patients are not aware of the availability of the rehabilitation service in the country. Prosthetic rehabilitation requires many sorts of materials and techniques to design good and functional device. Most of materials such metals, resin, plastic and silicone are commonly used, nevertheless they still present advantages and disadvantages. The technique described in this study reflects the aesthetic as well as the functional and comfort aspect of the finger prosthesis that meet the requirement of the user. It required a lot of expertise to rehabilitate distal amputation with silicone technology in a low resource country. Moreover, the present case involved multiple amputations of the fingers of both hands that make it quite difficult to treat. In addition, as there was also distal phalange amputation of the second finger of the right hand, the prosthesis made could not be longer as much as the normal length of a sound finger. The prosthesis made for the fingers of the left and right hands represent the first stage of the treatment. The next stage will tackle the remaining part of the left hand after surgical operation for correction of the third finger. Nowadays, with advanced technology, more functional bionic hands have been manufactured in developed countries. However, this technology is far yet to be implemented in a low resource country like Ghana. Using a technology that requires minimum cost and simple can increase a certain level of functionality and comfort of the patient.

## Conclusion

The rehabilitation of multiple-digit loss using real silicone cosmetic finger prostheses was one of the first attempts to fit a user in Ghana. Regaining hope and be socially comfortable is always a good wish for any amputee. In case of partial hand amputations, the aesthetic and functional aspects are very important. Using a technique that contributes to the patient´s quality of life needs to be considered. For better rehabilitative services and quality of life of patients, introduction of silicone technology into the training program in the country is highly recommended.
